# The Making of a Modern Hospital

**Published:** 1911-10-28

**Authors:** 


### THE MAKING OF A MODERN HOSPITAL.

### IV.—The Departments of the Modern Hospital.

So vast and complicated a machine as the modern hospital may claim to be cannot exist successfully unless some attempt is made to systematise its working according to certain definite rules of classification. When we compare its prototype, such as the old General Hospital at Vienna, with a type of to-day, such as the Johns Hopkins at Baltimore, we are at once struck by the fact that the main feature which renders the comparison all in favour of the modern example is this attempt at systematisation. The old institution, like some of our Poor Law Infirmaries of to-day, was a conglomeration, inchoate in its activities, restricted in its functions owing to a want of proper means to render available its resources, and cramped in its usefulness owing to the absence of system which led to constant overlapping. The modern institution, on the other hand, is a conglomeration of departments, each separate and independent so far as its working is concerned, but all interdependent so far as the whole is concerned, and working in harmonious unison owing to the fact that experience has gradually evolved a definite system which avoids overlapping and conduces to economy of energy.

### The Hospital of Yesterday.

These differences are well seen in comparing the institution of to-day with its analogy during the last century but one. The old Vienna General Hospital consisted of a large ward into which were admitted all sorts and conditions of patients, who were treated by the visiting physicians. When, as is so well described by the medical man who wrote the most concise and readable history of modern medicine, the importance of differentiating between surgical and purely medical patients was realised, the demarcation between the wards for the one group and those for the other was settled only after prolonged consideration. Finally this division into a surgical and a medical side (a division which seems so obvious to every hospital worker nowadays) was accomplished only after a struggle, since the physicians thought that there was no primary necessity for such a "special department" as a surgical ward! So to-day we find that many medical members of hospital staffs object to the creation of special departments which are rendered necessary by the rapid advances made in special lines of treatment.

We still see the influence of this antagonism on the part of the "general surgeon or physician" in the state of the special departments at some of our hospitals to which are appointed members of the general staff who are not specialists, but merely general consultants who have been selected, apparently, on the assumption that any member of the staff could, if he tried, conduct the department on successful lines. With this antagonistic influence and its unfortunate results we hope to deal at length in another chapter; for the present we only mention that it exists.

### And Its Modern Counterpart.

Following this division into a medical and surgical side came the creation of other departments, both administrative and technical. It became recognised gradually that it was conducive to economy both in energy and in money to make the institution self-producing as much as possible. The old institution contracted out for its laundry, its medicines, its surgical and medical appliances, its daily necessities, its repairs, its water, and its lighting. The modern hospital attempts, more or less successfully, to be independent of outside sources. It has established its own plant on the premises for the creation of adequate power to light its wards, to pump up its water, to heat its buildings, to wash its dirty linen, to sterilise its instruments, bandages, and dressings; it has developed its own dispensing department and acquired the means to manufacture many of its own medical supplies. All this has necessitated the creation of special departments, with the result that if one were asked to define in what lay the essential difference between the prototype and the modern institution there would be no hesitation in replying that it is in this departmentalisation, in a definite systematic way, of its energies.

### The Division into Departments.

The modern hospital, therefore, may be considered as a conglomeration of special departments, each in a sense independent, standing by itself and working along its own lines, but all mutually co-active, co-operant, and therefore interdependent. From its very foundation the institution is worked on departmental lines. The question of site, plan, and construction is a matter for the architects', the engineering, and the works departments, and so long as the institution exists these departments will have to exist, for their energies are always needed. The architects' department is concerned with the planning of improvements and reconstructions; it has to keep abreast of the discussions on hospital construction and planning if the institution to which it is attached is to have the benefit of modern developments in these subjects. The engineers' department will similarly have to deal with developments in machinery and plant; it will have to occupy itself with the ever-arising problems of drainage, ventilation, water supply, lighting, power plant, and special machinery. The works department is closely connected with these two departments; it acts in consultation and combination with them, and it needs a larger staff whose activities are concentrated on what may be called the structural upkeep of the hospital.

### The Administrative Side.

On the administrative side we have the secretary's or clerical department. This has to deal with the large mass of clerical work that the administration of a modern hospital brings with it. So enormous and so complicated is the work in this department at a large modern institution like the Johns Hopkins or the Virchow that the department has gradually become specialised in various ways, so that subsidiary working units have become necessary. These are the correspondence department, the finance department, the inquiry and almoners' department, the contract department, the medical school department, the funds department (which regulates the administration of the charitable and other funds under the control of the hospital), the chaplain's department, the matron's or housekeeper's department, the staff department, the nursing department, and the administrative department itself. The scope of these various special departments on the administrative side is sufficiently explained by the terminology. Thus the matron's department deals with the work of the nurses, more especially with the supervision of the nursing staff, with arrangements for their comfort and discipline; the housekeeper's department, on the other hand, may be considered subsidiary to that of the matron, and in most institutions is incorporated with it. In others, however, where the housekeeper is distinct from the matron, this department—also known as the steward's department—has nothing whatever to do with the nursing but concerns itself mainly with the supply of provisions for the institution. The staff employed in these departments is sometimes very large, and is generally known as the administrative staff, as distinct from the works' staff.

### Changes on the Medical Side.

Coming now to the medical department, it is at once noted that with the increase in size and usefulness of the institution it is almost inevitable that there should also be an increase in the number of special departments. The establishment of such departments during the last twenty-five years has perhaps been on a scale which is not warranted by the work that the departments, as such, have accomplished; but in view of the fact that most of them are concerned with special lines and methods of treatment rendered necessary by the advance in modern medical methods, it seems unreasonable to cavil at their multiplication, even when we may legitimately object to some of them. No institution that is up to date and progressive can afford to neglect new lines of treatment.

### Hospital "Experiments."

Our modern hospitals are experimental research institutions in the best sense of the term. We say this deliberately and advisedly, for the public still appears to think that no hospital is justified in "experimenting." It is true the public has a very erroneous idea of what is meant by the term "experiment." It presupposes that medical men in hospital practice try new drugs and new methods on their unsuspecting patients before they venture to try the same drugs or methods upon their private cases or upon themselves; moreover, it presupposes that such trials are essentially dangerous and a menace to the safety of the individuals who enter the hospital. There is no greater fallacy than this. A new drug or a new method of treatment evolved nowadays is the product of careful, patient, laborious and often self-sacrificing work on the part of the chemist or medical man; it is only when its usefulness has been demonstrated in the laboratory, when its dosage is known and its effects are ascertained, that it is employed in hospital practice. Its employment then, it is quite true, is in the nature of an experiment, but with the precautions taken nowadays such experimentation is the reverse of dangerous, while its results, so far as the public is concerned, are beneficial. The essential in hospital experimentation is that it is conducted under conditions which render whatever danger there may be infinitesimal. In hospital the patient is always under the observation of skilled attendants; whatever results are obtained are subjected to the skilled criticism of experts, none eager to accept without proof, few willing to relinquish an older method, with which they are well acquainted, for one that is yet on its trial. In that sense hospital experimentation is legitimate and justifiable, and it needs only a glance at certain of our modern methods of treatment to realise to the full what the public owes to such experimenting. Without it, for instance, Finsen would not have developed his method of treating lupus, nor Bier his method of treating tuberculous joints by means of passive hyperæmia, nor the phenomenal results with radium in the treatment of ulcerations and nævi. Hospital experimentation gave us bromide of potassium for the treatment of epilepsy, thyroid for the treatment of myxœdema, scarlet red for the treatment of granulating surfaces, and tuberculin for the diagnostic reactions associated with the names of Von Pirquet and Calmette. It has given us the posterior operation for gastro-jejunostomy, the means of restoring the motive power of a paralysed limb by anastomosing the nerves, new methods of detecting obscure but important lesions in deeply hidden organs, such as the pancreas, and without it, it is probable, that Lister's method of surgical procedure, which has revolutionised operative interference and reduced the mortality in the surgical wards from thirty-three per cent. to far less than eight per cent., would not have made such rapid advance as it has done.

In this sense, then, hospital experimentation needs no apology, and any institution may feel proud of being ranked as an experimental research institution, so long as this object is given its right—that is, a secondary—place, for the real aim and object of the institution is, after all, the cure and treatment of disease. That being the case, it is clear that the managers of such an institution are not justified in refusing to avail themselves of new methods of treatment and cure on the ground that these are yet in an experimental stage. The consideration of these new methods is imperative, and their trial, in the vast majority of cases, means the instalment of new appliances, and sometimes of an entirely new department. In the next article we will briefly outline the departments on the medical side which have been made necessary by recent advances in surgery and medicine.

(*To be continued.*)

